# A Genome-Wide Gene Expression Signature of Environmental Geography in Leukocytes of Moroccan Amazighs

**DOI:** 10.1371/journal.pgen.1000052

**Published:** 2008-04-11

**Authors:** Youssef Idaghdour, John D. Storey, Sami J. Jadallah, Greg Gibson

**Affiliations:** 1 North Carolina State University, Raleigh, North Carolina, United States of America; 2 Princeton University, Princeton, New Jersey, United States of America; 3 HRH Prince Sultan International Foundation for Conservation and Development of Wildlife, Agadir, Morocco; 4 University of Queensland, Brisbane, Queensland, Australia; Stanford University School of Medicine, United States of America

## Abstract

The different environments that humans experience are likely to impact physiology and disease susceptibility. In order to estimate the magnitude of the impact of environment on transcript abundance, we examined gene expression in peripheral blood leukocyte samples from 46 desert nomadic, mountain agrarian and coastal urban Moroccan Amazigh individuals. Despite great expression heterogeneity in humans, as much as one third of the leukocyte transcriptome was found to be associated with differences among regions. Genome-wide polymorphism analysis indicates that genetic differentiation in the total sample is limited and is unlikely to explain the expression divergence. Methylation profiling of 1,505 CpG sites suggests limited contribution of methylation to the observed differences in gene expression. Genetic network analysis further implies that specific aspects of immune function are strongly affected by regional factors and may influence susceptibility to respiratory and inflammatory disease. Our results show a strong genome-wide gene expression signature of regional population differences that presumably include lifestyle, geography, and biotic factors, implying that these can play at least as great a role as genetic divergence in modulating gene expression variation in humans.

## Author Summary

The incidence of complex diseases such as diabetes, asthma, and depression is almost epidemic in many countries and coincides with transition in lifestyles. Clearly this is a result of interaction between modern cultural and environmental factors with the genetic legacy of human history. To estimate the extent of the effects of environmental factors, including lifestyle and geography, on gene expression, we examined gene expression differentiation in peripheral blood leukocyte samples from three Moroccan Amazigh groups leading distinct ways of life: desert nomadic, mountain agrarian and coastal urban. Our data shows that as much as one third of the leukocyte transcriptome is associated with differences among the three regions. Network analysis implies that specific aspects of immune function are strongly affected by regional factors and may influence disease susceptibility. Genetic and methylation differentiation between the three regions is limited and is unlikely to explain the extent of the observed gene expression differentiation. Insight gained from this study highlights the impact transitions from traditional to modern lifestyles likely have on human disease susceptibility and further warrant the need to incorporate gene expression profiling alongside genetic association studies for the prediction of disease susceptibility.

## Introduction

Understanding the contribution of genetic and environmental factors to variation in gene expression in humans is essential to interpretation of the relationship between genotype and phenotype. Genetic differentiation has been the focus of several recent studies that have extensively mapped gene expression variation to specific genomic variants in lymphocyte samples [1] and Epstein-Barr virus-transformed lymphoblastoid cell lines [2],[3]. The contribution of environmental factors to variation in gene expression in humans has not yet been explicitly investigated. Here we test the hypothesis that environmental factors can generate significant transcriptional variation by contrasting peripheral blood gene expression among three regionally distinct samples of Moroccan Amazighs, who are a genetically relatively homogeneous human population. The Amazighs, also known as Berber people, occupy northwest Africa and are thought to represent a genetically relatively homogeneous human population [4],[5]. They lead distinct ways of life and occupy diverse physical geographic habitats across Morocco thus providing an excellent opportunity to monitor the impact regional differences in living circumstances have on gene expression and therefore physiology.

Peripheral blood is a readily accessible tissue sample that integrates environmental factors such as immune exposure, diet, and psychological state. We collected peripheral blood samples and isolated total leukocytes for gene expression profiling. We set out to establish the extent of the effect environmental factors relating to lifestyle and geographic differences have on immune expression profiles. Whole-genome genotyping and methylation profiling were used to estimate the extent of population structure and methylation differentiation in our sample as a proxy for their effect on the observed expression differentiation.

## Results/Discussion

**Figure 1:**
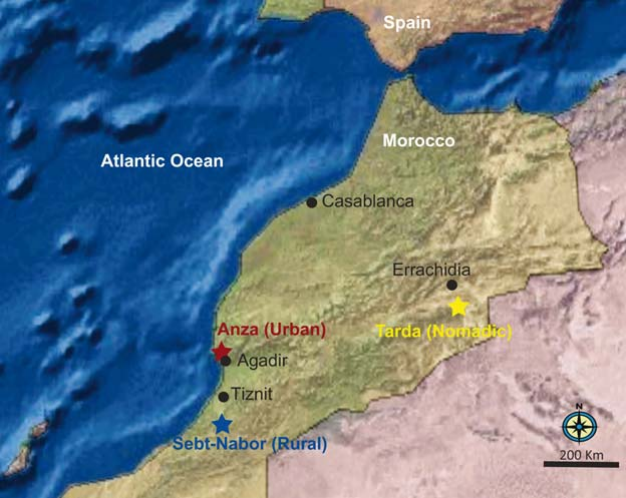
Geographic locations of sampled Amazighs groups in Morocco. A total of 52 peripheral blood samples were collected: 20 urban samples from the town of Anza (Latitude: 29.367°, Longitude: −9.633°), 15 rural samples from the rural village of Sebt-Nabor (Latitude: 31.450°, Longitude: −9.650°), and 17 nomadic samples from the Sahara desert in eastern Morocco (Latitude: 31.809°, Longitude −4.603°). Subsets of these were used in the gene expression profiling, genotyping and methylation profiling as described in Table S5.

Over a three week period, we obtained leukocyte samples from peripheral blood for gene expression profiling from 16 Bedouin living a traditional nomadic existence on the fringe of the Sahara desert near the town of Errachidia, 18 inhabitants of Anza, an urban slum-like settlement within the coastal city of Agadir, and 12 villagers from Sebt-Nabor, a remote rural mountain settlement south of Agadir (Figure 1). We isolated the total leukocyte population immediately after blood sampling [6], and extracted total RNA. Expression profiles were monitored with Illumina HumanRef8 v2 BeadChip oligonucleotide arrays representing over 22,000 annotated genes [7], 10,177 of which were expressed in the samples.

### Effect of Lifestyle and Geography on Gene Expression

We detected several distinct global profiles of expression, implying expression heterogeneity among individuals. This is seen in the analysis of all expressed genes, but is readily visualized in a heat map of two-way hierarchical clustering of the 1,000 most significant differentially expressed genes (Figure S1), highlighting the dominant impact that locality has on gene expression profiles. Surrogate variable analysis [8] was employed to capture and control for additional sources of expression heterogeneity that may include circadian cycle and age. This has been shown to increase the accuracy of analyses and avoid confounding of signals due to hidden sources of heterogeneity [8] (Figure S2 and Table S1). An analysis of variance including terms for region, sex, batch, and six significant surrogate variables confirmed the significant effect regional factors have on gene expression. A total of 3,725 of 10,177 (37%) of the expressed transcripts were differentially expressed with respect to region at the 1% false discovery rate (FDR) cutoff in a full three-way comparison, rising to 6,215 transcripts (61%) at 5% FDR. Table 1 shows the number of significant genes for each pairwise comparison. In the same analysis 30 transcripts were differentially expressed with respect to sex, at 1% FDR (70 at 5% FDR).

**Table 1:** Number of differentially expressed genes.

Significance Threshold	Urban *vs*. Rural	Urban *vs*. Nomadic	Nomadic *vs*. Rural	Male *vs*. Female
Bonferroni *P*<0.01	408 (4.0%)	103 (1.0%)	57 (0.56%)	22 (0.22%)
FDR<0.01	2,770 (27.2%)	1,069 (10.5%)	794 (7.8%)	30 (0.29%)
*P*<0.01	3,044 (29.9%)	1,897 (18.6%)	1,674 (16.4%)	365 (3.6%)

Numbers and percentages of differentially expressed genes at three different significance levels were obtained after fitting an analysis of variance model that included surrogate variables to account for unmodeled sources of differential expression, as well as fixed terms for location, sex, and batch.

**Figure 2:**
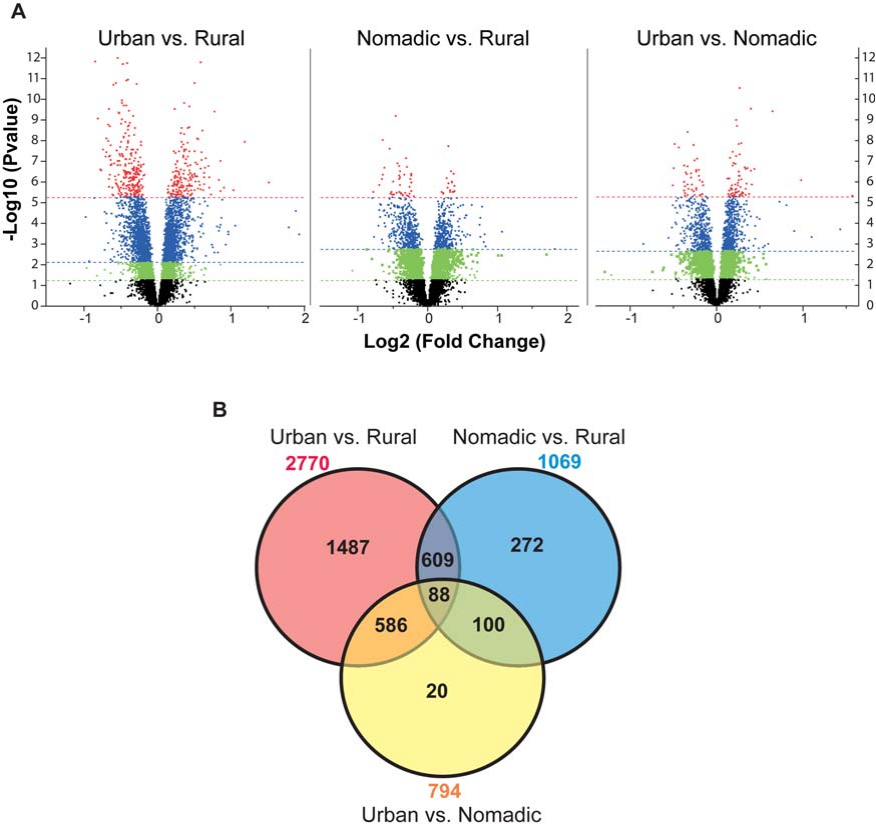
Volcano plots of statistical significance versus magnitude of differential expression between locations. (A) For each transcript, significance is shown as the negative logarithm of the *P* value on the y-axis, and the log base 2 of magnitude of mean expression difference is on the x-axis. Dashed lines indicate the threshold for significance (green: *P*<0.05, blue: 1% FDR, and red: Bonferroni adjusted *P*<0.05). The Venn diagram (B) shows the numbers of differentially expressed genes at 1% FDR for each comparison and the overlaps between them.

Given the geographical distance between the sampled localities (urban to rural: 150 km; urban to nomadic: 560 km; rural to nomadic: 650 km), and the scope of the differences in lifestyle, we expected the nomadic sample to be the most differentiated. However, volcano plots of significance against the magnitude of gene expression divergence for each pairwise comparison of regions (Figure 2A) imply that the nomads diverge less from both the rural and urban individuals, than the urban diverge from rural individuals. We also examined the differentiation of all three regions (Figure 2B), which shows that the majority of the differences between nomadic and rural individuals are also observed in contrasts of these two localities with the urban sample.

### Comparison of Environmental and Genetic Effects on Gene Expression

It is usually difficult to contrast the genetic and environmental contributions to leukocyte expression variation simultaneously, since different ethnic groups occupy different environments, but comparison with published results from laboratory cultures of cell lines implies that the regional effect is relatively large. Transcript abundance has recently been measured in B lymphoblastoid cell lines derived from individuals of Asian, African and European ancestry [9]–[11]. The proportion of genes differentially expressed was for example jointly estimated within and among populations [11], where it was concluded that approximately 17% of genes differentiate African and European populations. This percentage is based on the estimate that 83% of the genes are true nulls (that is, false positives if all genes are assumed to be significantly different) in the comparison. Using more stringent criteria, just 50 of 5,194 expressed genes were found to separate the two CEPH samples at an FDR of 20%, compared with 2,770 of 10,177 genes at an FDR of 1% in our similarly sized sample of leukocytes. In a reanalysis of the Asian and European comparison data set from ref. [12], it was shown that at least 94% of genes are differentially expressed with respect to a temporal batch effect and ancestry [13]. The reanalysis also estimated that 79% of genes are differentially expressed with respect to the batch effect among individuals of European ancestry. Therefore, of the 94% of genes differentially expressed with respect to year and ancestry, it can be estimated that 79% of these are due to the batch effect alone. This yields an estimate of 94%−79% = 15% of genes being differentially expressed with respect to ancestry, which is similar to that found in ref. [11], and approximately half the level of differentiation we observe due to regional non-genetic factors.

The effect of regional factors in our study is by contrast similar to that of genetic influences on breast tumors [14] and of HIV infection on peripheral blood [15]. We have reanalyzed the Affymetrix dataset in ref. [15] contrasting PBMC expression in HIV positive and negative mothers in a rural village in Botswana. HIV infection status significantly impacts expression of 31% of the 10,000 most strongly transcribed transcripts at an FDR of 1% in this study of a similar size. Consequently, expression heterogeneity on a scale similar or greater to that observed among Moroccan lifestyles can arise within a single community due to an identifiable environmental agent. Furthermore, reanalysis of a breast cancer cDNA microarray dataset [16] shows that 33% of transcripts distinguish BRCA1 from BRCA2 mutant tumors [14]. Thus, taken at face value, regional influences including lifestyle and geographic effects appear to make as large a contribution to leukocyte expression as common immune-related diseases.

It should be emphasized though that power to detect differential expression varies among studies, being heavily influenced by such technical factors as the array platform, sample size, and RNA handling methods, not to mention biological factors such as tissue homogeneity, genetic diversity, and individual environmental or cultural differences. Nevertheless, the studies contrasting lymphoblastoid lines from different populations should have been well powered to detect differences of the magnitude we attribute to lifestyle differences, and we also note that the estimates of the fraction of differentially expressed genes among lifestyles was not strongly affected by partial reduction of our dataset. Intriguingly, an estimate of heritability of transcription in PBMC samples from Mexican American families [1] was congruent with an estimate of heritability of transcription in transformed lymphoblasts from a British asthma cohort [3], both recent studies finding that over one quarter of transcripts have heritabilities in excess of 0.3. Heritability provides an estimate of the genetic contribution to the expression trait, but is by definition a characteristic of a single population in a common environment. Taken together these and other studies thus establish that there is ample genetic variation within populations, and that while there is some divergence between human ethnic groups, the potential impact of environmental variables can be much greater than that of genetic divergence.

### Genetic Differentiation Is Limited in our Sample

Although Amazighs are homogeneous relative to other human groups [4], having retained a distinct cultural identity in Morocco alongside Arabs and sub-Saharan Africans in the face of repeated invasions throughout recorded history, it is possible that there has been modest genetic differentiation among the three communities [5]. Whole genome genotype profiles [17] were obtained for eight randomly selected individuals from each region and principal component analysis was used to infer the extent of genetic variation from over 300,000 autosomal SNPs [18]. Out of the top 10 axes of variation, only one is significant (*P* = 0.0006, Tracy-Widom test). It distinguishes three residents of urban Anza, possibly indicating recent Arab admixture (Figure S3 and Table S2). No significant axes of variation were detected when these three individuals were removed from the analysis. There was a suggestion of genetic divergence of the nomads in the second axis but it only explained a small fraction of the genotypic variation and was only marginally significant (*P* = 0.0167). By contrast, application of principal component analysis to a European American sample [18] indicated that the top ten axes were statistically significant (*P*<0.01 for each) with the top two axes having *P*<10^−12^, supporting the evidence that Moroccan Amazighs are a homogeneous group relative to other human groups.

The absence of meaningful population structure was also confirmed with *Structure* Version. 2.2 [19]. We applied the program to 11,000 autosomal SNPs (500 randomly selected and approximately uniformly distributed from each of the 22 autosomal chromosomes) at *K* = 2–3 (Figure S4). At *K* = 2, *Structure* first separates three individuals (8D, 5D, and 5G in Figure S1) from the rest of samples; the same individuals were distinguished by *Eigenstrat* analysis on the significant axis of variation. At *K* = 3, two nomadic individuals cluster as a distinct unit but the rest of samples show high membership coefficient to one group. Increasing the number of SNPs had no effect on the results. As *K* is increased, pairs of individuals show evidence for relatedness, but these are not members of the same population, further confirming the relative absence of population structure in the dataset. Comparison of pairwise differences among individuals confirms that, excluding the three outliers in Anza, there is no notable difference in degree of relatedness of individuals within the different populations. Taken together these results indicate that the majority of the divergence in gene expression described here is unlikely to be explained by genetic divergence.

Despite the absence of genomewide differentiation between the study sites, it is possible that some of the expression divergence could be due to genetic differences at a small number of loci that regulate hundreds of downstream transcripts. These would likely need to be nearly fixed between the populations to cause the almost complete correspondence between expression profile and locality. To explore this possibility, we estimated *Fst* for each of over 300,000 sites for each of the three pairwise comparisons of populations, and plotted the values in a sliding window of 100 sites along each chromosome. *Fst* values are typically below 0.05 but occasionally range up to just 0.11, averaging between 0.043 and 0.052 in each comparison, again confirming the low level of genetic divergence. No fixed differences were detected.

To explore the relationship between genetic and expression divergence at each locus, gene-specific *Fst* measures were calculated by averaging *Fst* values of all SNPs located within 1-Mb upstream and downstream of the expression probe. No correlation between *Fst* and expression fold change (Figure S5A) or significance (Figure S5B) was detected when this analysis was performed for all 10,177 expressed genes and for all three pairwise comparisons. Although some *cis*-acting, presumably regulatory, variants have been shown to have large effects on transcript abundance [1]–[3], this result argues against the population expression differences being attributable to genetic divergence at thousands of locally acting polymorphisms in our study. While a few genes show both genetic and expression divergence, their number is no greater than expected by coincidence.

### Differential Methylation Analysis

**Figure 3:**
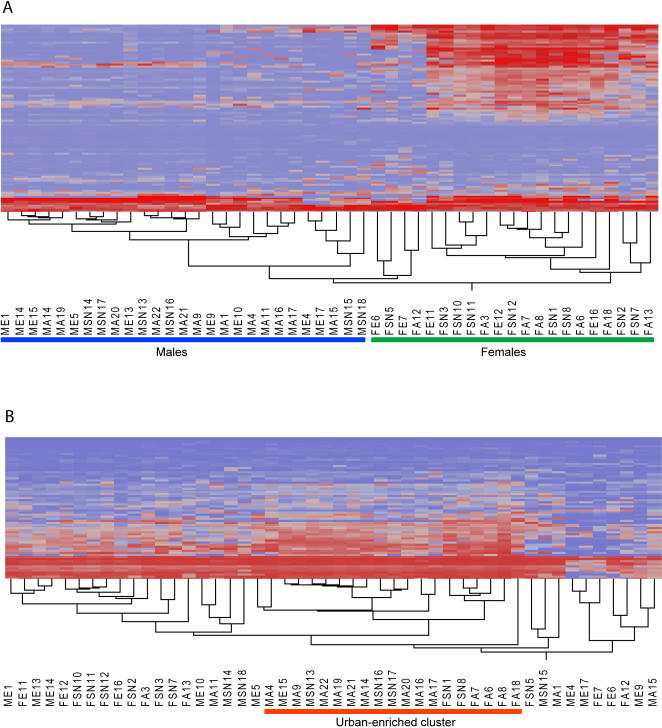
Sex and location effects on methylation patterns. Two-way clustering of differentially methylated CpG sites at *P*<0.05 (ANOVA) for the sex effect (A) and for the location effect (B). Sample labels indicate their sex and location (M: male, F: female, E: nomad, SN: rural, and A: urban). There is clear separation of the sexes in (A), and a suggestion of a signature of urban living for a dozen or so genes in (B) highlighted as the Anza-enriched cluster.

The observed differential expression could be due to differences in the ratios of the numerous cell types present in the total leukocyte population, to transient changes in activity of transcription factors and micro RNAs, or to longer-term epigenetic modification of chromatin structure. To test for one possible epigenetic mechanism, we measured the degree of methylation at 1,505 CpG sites [20] representing 805 genes of various classes, including tumor suppressor genes, oncogenes, genes involved in DNA repair, cell cycle control, differentiation and apoptosis. 420 of these sites were within genes included in our list of transcripts differentially expressed among locations. 58 differences in CpG site methylation between males and females were detected at a *P*<0.01 with a mixed model analysis of variance (Figure 3A), the majority involving X-linked genes, as expected given the correspondence of methylation with X-inactivation [21] (Figure S6), thus providing a positive control for the methodology. However, only 18 CpG sites were found to differ between regions in this analysis at the same significance level, which is no more than expected by chance. A small signature of a dozen or so genes differentiated half of the urban population (Figure 3B), but on the basis of our results epigenetic modification via methylation can only account for a small fraction of the expression divergence between regions.

### Expression Differences and Impact on Immune Function

**Figure 4:**
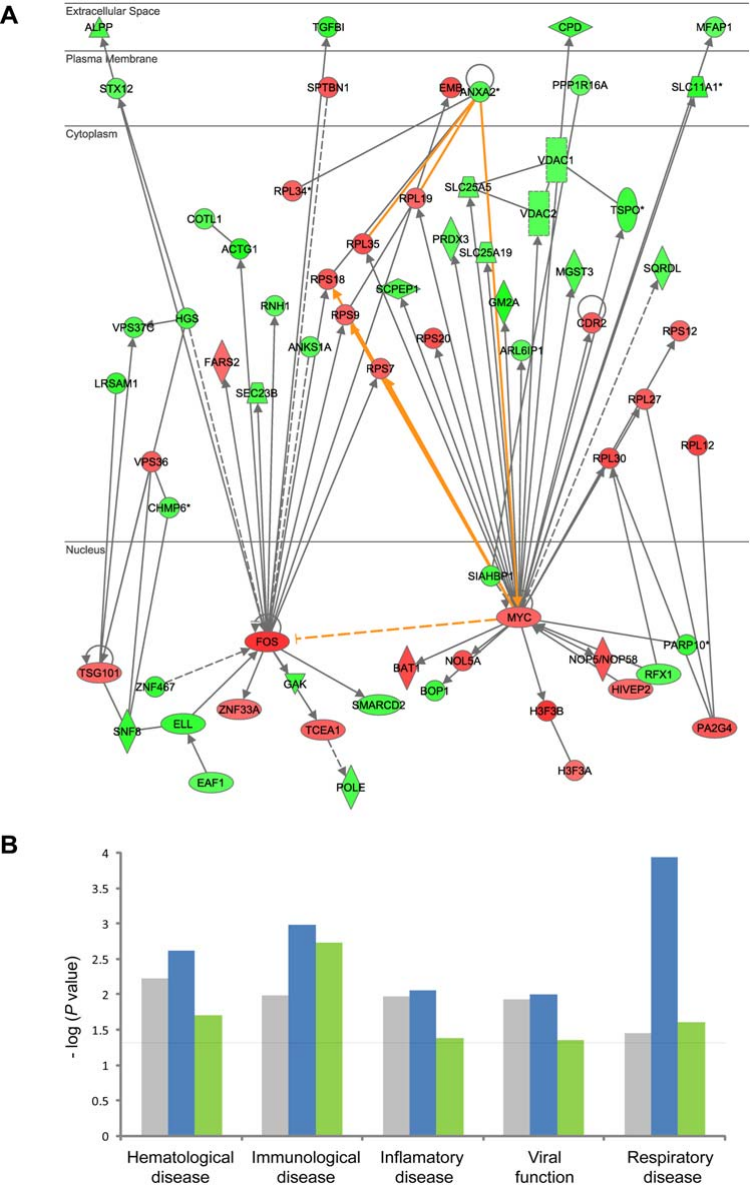
Functional analysis of differentially expressed genes. (A) Differential expression of the FOS and MYC networks and enriched disease classes. The Ingenuity Pathways Knowledge Base (IPKB) was used to generate networks of interacting genes that are overrepresented in the set of transcripts differentially expressed (based on a 1% FDR cutoff) between the urban and rural samples. The top two networks are focused on the FOS and MYC transcription factors, and every one of the genes that the IPKB indicate as interacting either genetically or biochemically are differentially expressed in this comparison. Network connectivity is indicated as solid edges for direct interactions, and dashed edges for indirect interactions. Transcripts are displayed in green for down-regulated and red for up-regulated, while cellular compartments in which the gene products are localized are also indicated. Gold edges highlight shared interactions. The list of genes, their fold change and *P* values are listed in Table S3. (B) Overrepresentation of disease classes affected by differentially expressed genes. Some of the Ingenuity Knowledge Database disease bio-function categories enriched (*P*<0.05) in differentially expressed transcripts (1% FDR) in the three lifestyle pairwise comparisons (grey, urban *vs*. rural; blue, nomadic *vs*. urban; green, nomadic *vs*. rural). Fisher's exact test was used to calculate the *P* value associated with the probability that the number of genes in each biological function and/or disease assigned to that data set is greater or less than expected by chance given the numbers of genes expressed in leukocytes.

Global comparison of differential expression according to gene ontology classes and pathway analysis implicates divergence in core immune competence among the three groups. Ingenuity Pathway Analysis was used to explore connectivity among differentially expressed transcripts and identified significant differential expression networks anchored by key immunoregulatory factors. For example, all of the genes connected to the cell cycle and apoptosis regulators FOS and MYC [22] show significant effects of regional factors (Figure 4A and Table S3). The identities of the most differentially expressed genes also suggest that the expression divergence among the three Amazigh localities is likely to impact immune system function and disease susceptibility (Fig 4B and Table S4). ). The following specific examples illustrate this conclusion, which is further corroborated by network analysis of clusters of differentially expressed genes.

The most enriched Ingenuity Knowledge Database disease category in the differentially expressed genes list is the respiratory disease class (*P*<0.0001, Fisher's exact test). Our data shows that IL-8, a respiratory cytokine and genetic variants of which have been associated with asthma [23],[24], is dramatically upregulated in the inhabitants of urban Anza compared to the rural villagers (*P*<0.0005, ANOVA) and the nomads (*P*<0.0004). Additionally, four IRAK interleukin receptor-associated kinases, one of which is associated with respiratory illness [25], were differentially expressed by location (Figure S7). The urban Anza residents are exposed to pollutants from a nearby concrete manufacturing plant and other industrial units, and to viral pathogens that are not experienced by nomads or villagers, and are known to have an enhanced incidence of respiratory illness. This result suggests that a genotype-by-environment effect resulting in aberrant expression of IL-8 transcript abundance in leukocytes, should be considered in parallel with the use of genotypic variation as a possible diagnostic marker for respiratory illness.

The Human Leukocyte Antigen (HLA) complex is well known to mediate response to infection as well as autoimmunity, as it encodes the major histocompatibility molecules that present antigens to the immune system. Intriguingly, two non-classical members of the HLA complex, HLA-DM and HLA-DO, were differentially expressed among the urban population compared to the rural villagers (*P*<0.000009 and *P*<0.002, respectively) and to the nomads (*P*<0.0009 and *P*<0.006, respectively). These are thought to act in concert to process antigens for binding to MHC [26], and are strongly co-regulated across our sample. This result warrants further exploration of association of these genes with immunological disease.

One of the two most divergent transcripts encodes POU2F2 (also known as OCT2), the octamer-binding transcription factor that regulates differentiation of leukocytes [27]. Transcription of this gene tends to be elevated in villagers relative to the other two groups. ELK1, another important lymphoblastoid transcription factor [28] that regulates FOS transcription [29], showed elevated expression only in individuals from the village. Potential and validated targets of ELK1 are enriched (*P*<0.004, Fisher's exact test) in the list of differentially expressed genes as determined by counts of putative binding sites listed in the TRANSFAC database [30]. Consequently, modulation of expression of this single regulator probably has pleiotropic effects on immune function, both at the level of cellular differentiation and function.

### Conclusion

The most plausible explanation for the dramatic differentiation of as much as a third of the transcriptome described here is that a combination of biotic, abiotic, and cultural differences is involved. A significant portion of these environmental factors can be attributed to aspects related to lifestyle such as nutrition, history of immune exposure, and psychological stress. Consequently, insight gained from this study highlights the impact transitions from traditional to modern lifestyles likely have on human disease susceptibility, particularly through their impact on immune function. We speculate that diseases due to genetic factors in urban populations may bear little resemblance to the impact of the same genetic factors in traditional societies. Since the causal environmental factors and the mechanisms through which they act remain to be identified, we advocate the incorporation of gene expression profiling alongside genetic association studies for the prediction of disease susceptibility.

## Materials and Methods

### Study Populations and Collection Protocol

Ethical approval for the study was granted by the Institutional Review Board for the Use of Human Subjects in Research at North Carolina State University, and the Moroccan Ministry of Health. Peripheral blood samples were collected under informed consent from 52 self-reported Moroccan Amazigh. All subjects were between the ages of 18 and 52 and were reported to be in good health at the time of blood sampling. The pastoral nomadic subjects of the study (average age = 35) are Bedouins from an Amazigh tribe inhabiting a region known as Tarda (Latitude: 31.809°, Longitude −4.603°) in the Sahara desert. The Bedouins live in traditional tents and their subsistence comes from domesticated animals. All but three subjects are unrelated (in Figure S1, individuals labeled 9A and 6E are siblings and 8E is their cousin). The rural subjects (average age = 32) are unrelated permanent residents of the Sebt-Nabor village (Latitude: 31.450°, Longitude: −9.650°). The subjects have a traditional lifestyle based on traditional agriculture and herding with very little exposure to urbanized lifestyle, one indication of which is that access to the village was by a four hour donkey ride. The urban subjects of the study (average age = 31) are unrelated permanent residents of the urban town of Anza (Latitude: 29.367°, Longitude: −9.633°). All the subjects have been living in Anza for at least the last 10 years.

The same sample collection protocol was followed for the three collection sites in order to minimize the effect of this source on gene expression heterogeneity. Blood samples were collected within a period of three days for each locality in December 2006, with collection each day spread over a 6 hour period from mid-morning to mid-afternoon. Approximately 15 ml of peripheral whole blood was collected by a phlebotomist using venipuncture. The total leukocyte population was immediately (within 2 minutes) isolated from 9 ml, and its total RNA was stabilized with the LeukoLOCK™ Total RNA Isolation System (Ambion, Austin, TX). The system incorporates depletion filter technology to isolate leukocytes and eliminate plasma, platelets, and red blood cells, and RNA*later* to stabilize the RNA in the cells captured in the filter. The remaining blood (approximately 6 ml) was stored in EDTA tubes for DNA extraction. The filters and blood samples were kept on ice, and then frozen at −20°C within three days of collection. While we cannot exclude the possibility that sampling differences at each locality contribute to expression divergence, the fact that several individuals cluster outside their locality (Figure S1) argues against this.

### RNA and DNA Preparation

Total RNA extraction, cDNA and cRNA synthesis were performed with the Illumina TotalPrep RNA Amplification kit (Ambion, Austin, TX) following the manufacturer's instructions. Size distribution of the extracted total RNA and the amplified cRNA was checked with Agilent's *RNA 6000 Nano LabChip* kit and *2100 Bioanalyzer* (Agilent Technologies, Palo Alto, CA). The quality of RNA and cRNA was comparable among all samples used further in the experiment. DNA samples were extracted with QIAamp DNA Kit (Qiagen, Valencia, CA). Standard gel electrophoresis and the ND-1000 (NanoDrop Technologies, Wilmington, DE) were used to check DNA quality and quantity, respectively. The list of samples used for each experiment described below is available in Table S5.

### Gene Expression Profiling

Illumina's HumanRef-8 v2 BeadChips (Illumina, San Diego, CA) were used to generate expression profiles of more than 22,000 transcripts with 500 ng of labeled cRNA for each sample and following manufacturer's recommended protocols. All expression data are available at NCBI Gene Expression Ominbus (GEO) under series number GSE8847. The individual expression arrays are listed as GSM219988 through GSM220033. An Excel spreadsheet list of all differentially expressed gene is also available online at the PLoS Genetics website as Dataset S1, and significance criteria for all transcripts are provided as Dataset S2.

A randomized design was used to minimize chip effects. Four individuals were replicated in the two batches; these clustered adjacent to one another in hierarchical analysis and the expression intensities were averaged in the statistical analysis. Expression intensity measures were obtained from an average of 30 beads for each transcript. The BeadChips were imaged with an Illumina BeadArray Reader. The raw intensities were extracted with the Gene Expression Module in Illumina's BeadStudio software. Expression intensities were log2 transformed and median-centered by subtracting the mean value of each array from each intensity value. 10,177 transcripts with expression at or above background levels averaged across all the arrays were retained for further analyses. These represent transcripts remaining after removal of 12,000 bead measurements that were considered to lay below background detection levels because they are less than the inflection point of a plot of rank-ordered log2 transformed, median-centered, intensities of all of the transcripts on the array. A median-centered normalization was carried out again from the raw intensities considering only the 10,177 most expressed transcripts, and the resulting relative fluorescence intensities were used in further analyses. List of transcripts considered expressed in leukocytes and lists of significance for differential expression for each comparison are available in Tabes S6 and S7, respectively.

### Statistical Modeling of Gene Expression

It has recently been shown that if there are additional sources of expression variation due to factors not included in the model, then this can lead to unreliable differential expression analyses due to large-scale dependence among genes and potential confounding with these unmodeled factors [8]. “Surrogate Variable Analysis” was developed to directly use the known variables (here, Location, Sex, and Batch) and the entire expression data set in order to estimate the signatures of these unmodeled factors, called “surrogate variables”. Table S1 lists the surrogate variables estimated and utilized in all of the analyses. After identifying surrogate variables, differential expression was estimated using an analysis of variance following standard methods [31] on the basis of the following model:
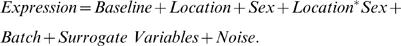



For each of the *Location* and *Sex* variables, a *P* value (ANOVA) measuring significance of differential was obtained for each gene. False discovery rates were calculated according to the qvalue software package [14]. It should be noted that even though the surrogate variables represent random effects, we were able to effectively treat them as fixed in the model fitting process, since all inference was performed conditional on the surrogate variables in a conditional likelihood framework [32]. The effect on *P* value distributions when including surrogate variables is shown in Figure S2.

### Functional and Promoter Enrichment Analysis

The network, functional and biomarker analyses were generated through the use of Ingenuity Pathways Analysis. Genes whose expression was significantly differentially regulated between the three locations were included using a 1% false discovery rate cutoff from the surrogate variable analysis results. Fisher's exact test was used to calculate a *P* value associated with the probability that the number of genes in each biological function and/or disease assigned to that data set is greater or less than expected by chance given the numbers of genes expressed in the leukocytes. The program *PRIMA* [33] integrated in the software *Expander* [34] was used to find transcription factors whose binding sites are more frequent than expected by random in the promoters (spanning from 1000 bp upstream the TSS to 200 bp downstream the TSS) of the differentially expressed genes between locations. We used version 27 of the list of binding sites in the *TRANSFAC* database [30].

### Genotyping and Population Structure

Twenty-four samples (eight randomly selected samples from each population) were assayed with Illumina's Infinium HumanHap300 SNP Chip following standard procedures. The HumanHap300 SNP Chip contains over 318,000 SNPs derived from phase I of the International HapMap project [35]. The BeadChips were imaged using Illumina's BeadArray Reader and genotype calls extracted with the Genotyping Module in Illumina's BeadStudio software. Principal component analysis was used to infer the extent of genetic variation from over 300,000 autosomal SNPs using *Eigenstrat* as described in ref. [18]. *Structure* Version 2.2 [19] was used to infer population structure. We applied the program to 11,000 autosomal SNPs (500 randomly selected and approximately uniformly distributed from each of the 22 chromosomes) at *K* = 2–3. We used a model with admixture and correlated allele frequency for 100,000 iterations after a burn-in length of 20,000. We used small *K* to analyze population structure given our prior knowledge about the Moroccan population. *Structure* runs at *K* = 2–3 were repeated under the no admixture model with either correlated or uncorrelated allele frequencies with similar results.

### Methylation Assay

A methylation profile was obtained for 96 samples (14 nomadic samples, 15 rural samples, and 19 urban samples, each sample represented by two technical replicates) with Illumina's GoldenGate methylation Cancer Panel I array-based assay. DNA samples were first subjected to a bisulfite conversion reaction using ED DNA methylation kit (Zymo Research, Orange, CA) and then subjected to Illumina's GoldenGate methylation assay [20]. The GoldenGate Methylation Cancer Panel I spans 1,505 CpG loci selected from 807 genes falling into various classes, including tumor suppressor genes, oncogenes, genes involved in DNA repair, cell cycle control, differentiation and apoptosis. The raw methylation ratios were extracted using the Methylation Module in Illumina's BeadStudio after a background normalization that subtracts a background value derived by averaging the signals of built-in negative control bead types. A mixed model analysis of variance was applied on a CpG site-by-CpG site basis with the PROC MIXED procedure implemented in SAS ver 9.1 (SAS Institute, Cary, NC):
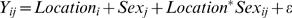



*Location* and *Sex* were considered fixed effects with the *i*th location (*i = * urban, rural or nomadic) and the *j*th sex (*j* =  male or female). The effect of interaction between *Location* and *Sex* was included in this model, and the error *ε* was assumed to be normally distributed with mean zero.

## Supporting Information

Figure S1 Two-way hierarchical clustering of expression. The heat map shows the clustering of expression profiles largely by location. Each row represents one of the top 1,000 transcripts for significance of the location effect and each column represents one individual. Intensity of red indicates relatively high expression relative to the sample mean, of blue relatively low expression. Individuals are identified by a code with the first letter representing gender (Male or Female), the second letter population (D, desert/nomadic; A, Anza/urban; V, Sebt-Nabor village/rural), the number corresponds to Illumina BeadArray, and last letter is a unique identifier within each array. Note that the highest level of clustering tends to be by population, while the two genders also tend to cluster within populations. The clustering was generated with Ward's method in JMP Genomics ver. 3.0 implemented in JMP ver. 7.0.
(0.43 MB PDF)

Figure S2 Effect of surrogate variable inclusion on significance testing. Quantile-quantile plots of the P-values resulting from differential expression analyses with and without SVA. The solid dots represent P-value quantiles and the dashed line is the line of equality. Curves above the diagonal imply larger P-values without SVA, and hence a gain in power from the analysis that include surrogate variables. (A) Urban vs. Rural vs. Nomadic. (B) Urban vs. Rural. (C) Urban vs. Nomadic. (D) Nomadic vs. Rural.
(0.11 MB PDF)

Figure S3 Eigenstrat analysis of population structure. The plot shows the first two eigenvectors of genotypic variance for the three Moroccan populations (red, urban; blue, rural; brown, nomads). Only the first eigenvector is significant (P<0.0006, Tracy-Widom test); it distinguishes three individuals within the urban sample as indicated in the figure. The second eigenvector suggests a separation of the nomads and villagers, but it is only marginally significant (P<0.0167) and explains only a very small proportion of the genotypic variance. Each square represents one of the 24 individuals who were genotyped.
(0.08 MB PDF)

Figure S4 Structure analysis of genotypic variation. Each individual is represented by a column that is partitioned into K colored segments representing the proportion of ancestry (Q value) from each of the K clusters for each individual using 11,000 autosomal SNP markers. Two Structure run at K = 2 and K = 3 are shown. At K = 3, 80% of individuals have high membership coefficient to one cluster.
(0.15 MB DOC)

Figure S5 Genomewide genetic and gene expression differentiation. Average Fst measures plotted against log2 fold change (A) and negative log10 probability resulting from differential expression analyses with SVA (B), for each of the 10,177 expressed genes and for each population pairwise comparison (Green, Nomadic vs. Rural; Blue, Nomadic vs. Urban; Red, Urban vs. Rural). Each open circle represents a gene. Gene-specific Fst values were calculated for each gene by averaging Fst values for all segregating SNPs within the gene and approximately 1Mb upstream and downstream the gene. The percent variance explained is less than 0.001 with p>0.05 for all six regressions represented by these plots.
(0.17 MB PDF)

Figure S6 Results of methylation analysis. Histograms show the distribution by chromosome of CpG sites of (A) the 1,505 CpG sites represented on Illumina's GoldenGate Methylation Cancer Panel I array; (B) the 97 differentially methylated CpG sites for the sex effect at P<0.05 (ANOVA); (C) the 69 differentially methylated CpG sites between locations at P<0.05, and (D) the 24 differentially methylated CpG sites for the sex and location interaction effect at P<0.05. The X chromosome is shown in dark green. Panel B can be considered a positive control for the success of the analysis since methylation is known to preferentially mark X-linked loci.
(0.14 MB PDF)

Figure S7 Correlations among Interleukin signaling components. The three plots show the average relative transcript abundance (log base 2 scale) for the indicated genes in the Urban (U: Anza), Nomadic (N: Bedouin), and Rural (R: Sebt-Nabor) locations. P values in brackets indicate the significance of the 3-way location term from mixed model ANOVA. Across all 46 individuals, IL-8 is negatively correlated with IRAK2 (P = 0.0008), IRAK1 is negatively correlated with IRAKBP1 (P = 0.008), and IRAK3 is positively correlated with IRAK4 (P = 0.004).
(0.08 MB PDF)

Table S1 Surrogate variables
(0.05 MB XLS)

Table S2 Eigenstrat principal component statistics
(0.06 MB PDF)

Table S3 List of genes in FOS and MYC networks shown in Figure 4A
(0.09 MB PDF)

Table S4 Top 15 networks generated by Ingenuity from the list of genes differentially expressed (1% FDR) for each location pairwise comparison
(0.11 MB PDF)

Table S5 List of individuals and analyses
(0.09 MB PDF)

Dataset S1 List of differentially expressed genes and their annotation.
(1.49 MB XLS)

Dataset S2 List of significance of differential expression by various criteria.
(6.83 MB XLS)
