# Supplementary material for: A Genome-Wide Gene Expression Signature of Environmental Geography in Leukocytes of Moroccan Amazighs
Source: PLoS Genet. 2008 Apr 11;4(4):e1000052. doi: 10.1371/journal.pgen.1000052 (PMC2290968; doi:10.1371/journal.pgen.1000052)

Figure S1. **Two-way hierarchical clustering of expression**. The heat map shows the clustering of expression profiles largely by location. Each row represents one of the top 1,000 transcripts for significance of the location effect and each column represents one individual. Intensity of red indicates relatively high expression relative to the sample mean, of blue relatively low expression. Individuals are identified by a code with the first letter representing gender (Male or Female), the second letter population (D, desert / nomadic; A, Anza / urban; V, Sebt-Nabor village / rural), the number corresponds to Illumina BeadArray, and last letter is a unique identifier within each array. Note that the highest level of clustering tends to be by population, while the two genders also tend to cluster within populations. The clustering was generated with Ward's method in JMP Genomics ver. 3.0 implemented in JMP ver. 7.0.

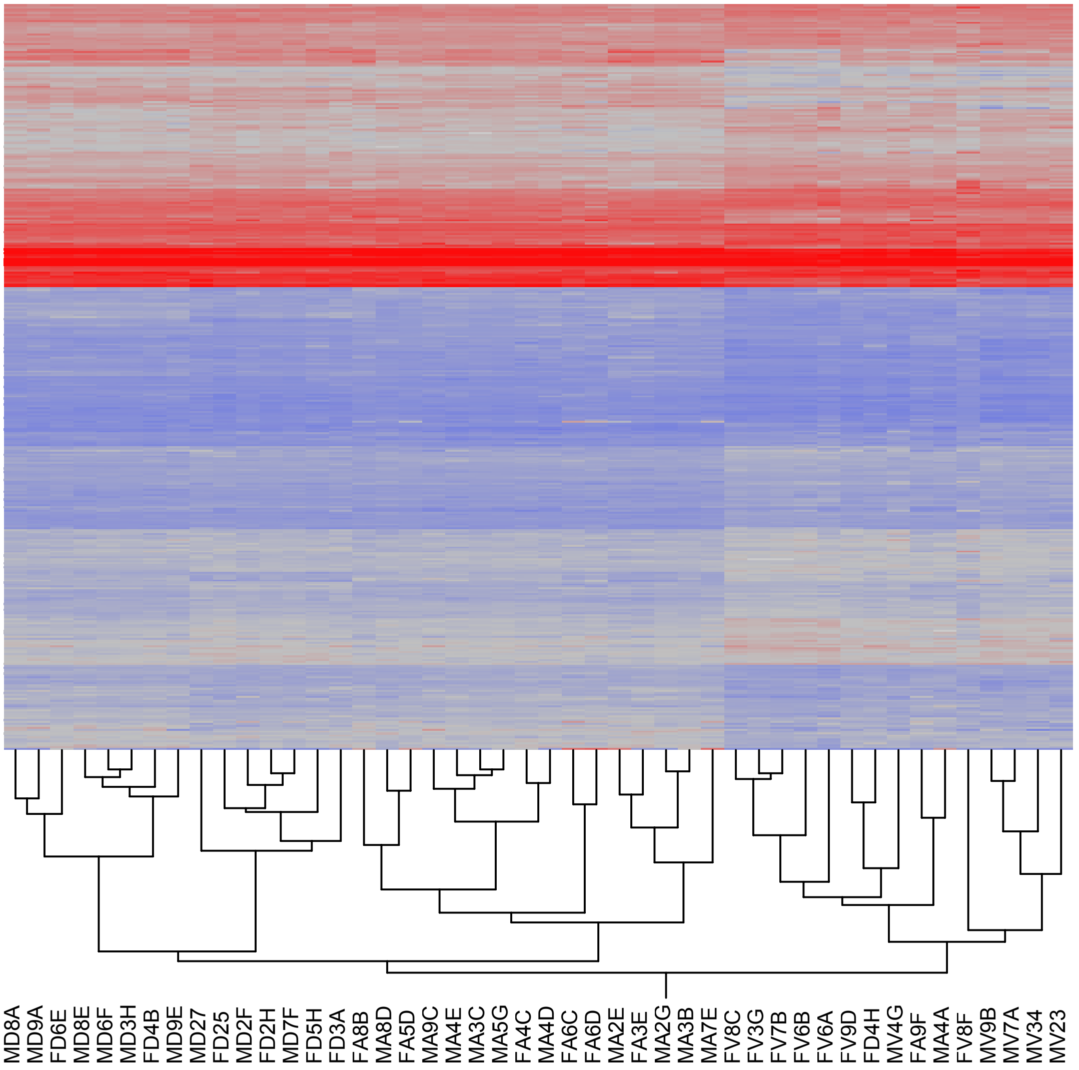

Supplement: Figure S1 — Two-way hierarchical clustering of expression. The heat map shows the clustering of expression profiles largely by location. Each row represents one of the top 1,000 transcripts for significance of the location effect and each column represents one individual. Intensity of red indicates relatively high expression relative to the sample mean, of blue relatively low expression. Individuals are identified by a code with the first letter representing gender (Male or Female), the second letter population (D, desert/nomadic; A, Anza/urban; V, Sebt-Nabor village/rural), the number corresponds to Illumina BeadArray, and last letter is a unique identifier within each array. Note that the highest level of clustering tends to be by population, while the two genders also tend to cluster within populations. The clustering was generated with Ward's method in JMP Genomics ver. 3.0 implemented in JMP ver. 7.0. (0.43 MB PDF) [file pgen.1000052.s001.pdf]
