# Supplementary material for: A Genome-Wide Gene Expression Signature of Environmental Geography in Leukocytes of Moroccan Amazighs
Source: PLoS Genet. 2008 Apr 11;4(4):e1000052. doi: 10.1371/journal.pgen.1000052 (PMC2290968; doi:10.1371/journal.pgen.1000052)

Figure S2. Effect of surrogate variable inclusion on significance testing. Quantile-quantile plots of the *P*-values resulting from differential expression analyses with and without SVA. The solid dots represent *P*-value quantiles and the dashed line is the line of equality. Curves above the diagonal imply larger *P*-values without SVA, and hence a gain in power from the analysis that include surrogate variables. (**A**) Urban *vs*. Rural *vs*. Nomadic. (**B**) Urban *vs*. Rural. (**C**) Urban *vs*. Nomadic. (**D**) Nomadic *vs*. Rural.

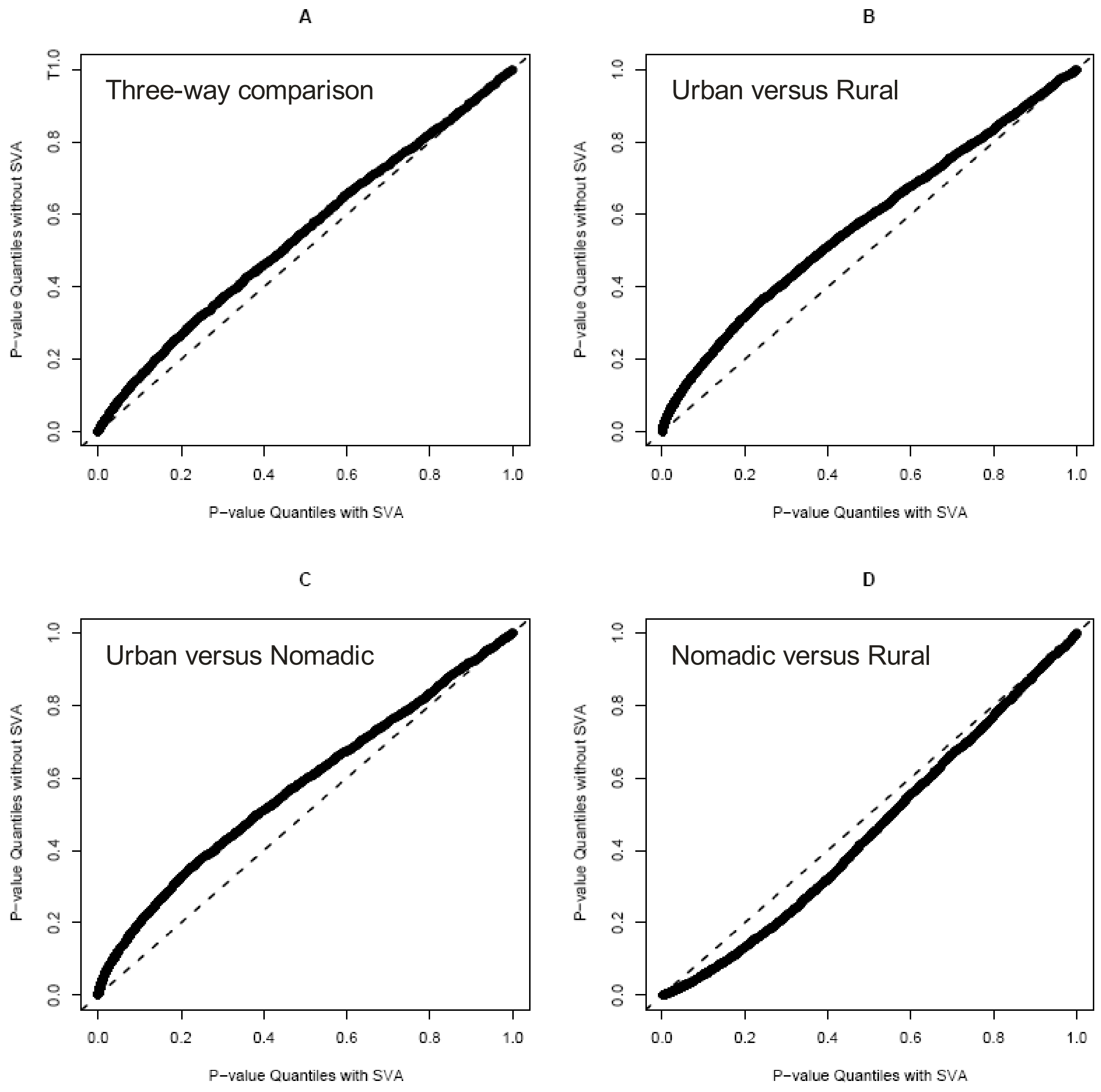

Supplement: Figure S2 — Effect of surrogate variable inclusion on significance testing. Quantile-quantile plots of the P-values resulting from differential expression analyses with and without SVA. The solid dots represent P-value quantiles and the dashed line is the line of equality. Curves above the diagonal imply larger P-values without SVA, and hence a gain in power from the analysis that include surrogate variables. (A) Urban vs. Rural vs. Nomadic. (B) Urban vs. Rural. (C) Urban vs. Nomadic. (D) Nomadic vs. Rural. (0.11 MB PDF) [file pgen.1000052.s002.pdf]
