# Supplementary material for: A Genome-Wide Gene Expression Signature of Environmental Geography in Leukocytes of Moroccan Amazighs
Source: PLoS Genet. 2008 Apr 11;4(4):e1000052. doi: 10.1371/journal.pgen.1000052 (PMC2290968; doi:10.1371/journal.pgen.1000052)

Figure S3. ***Eigenstrat* analysis of population structure**. The plot shows the first two eigenvectors of genotypic variance for the three Moroccan populations (red, urban; blue, rural; brown, nomads). Only the first eigenvector is significant ($P < 0.0006$, Tracy-Widom test); it distinguishes three individuals within the urban sample as indicated in the figure. The second eigenvector suggests a separation of the nomads and villagers, but it is only marginally significant ($P < 0.0167$) and explains only a very small proportion of the genotypic variance. Each square represents one of the 24 individuals who were genotyped.

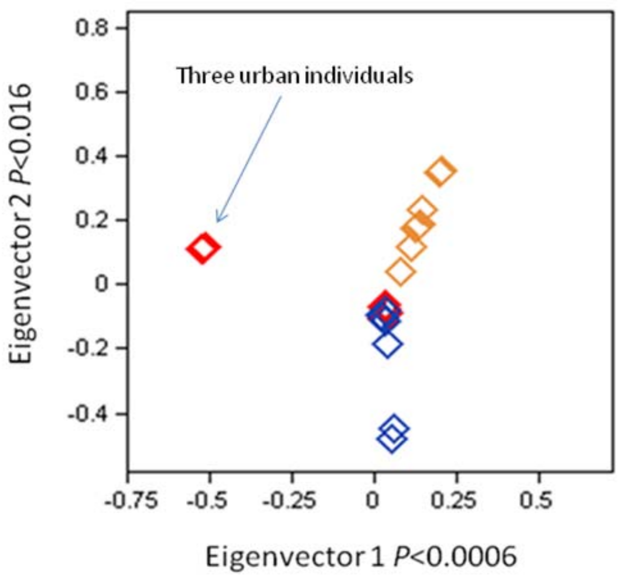

Supplement: Figure S3 — Eigenstrat analysis of population structure. The plot shows the first two eigenvectors of genotypic variance for the three Moroccan populations (red, urban; blue, rural; brown, nomads). Only the first eigenvector is significant (P<0.0006, Tracy-Widom test); it distinguishes three individuals within the urban sample as indicated in the figure. The second eigenvector suggests a separation of the nomads and villagers, but it is only marginally significant (P<0.0167) and explains only a very small proportion of the genotypic variance. Each square represents one of the 24 individuals who were genotyped. (0.08 MB PDF) [file pgen.1000052.s003.pdf]
