# Supplementary material for: A Genome-Wide Gene Expression Signature of Environmental Geography in Leukocytes of Moroccan Amazighs
Source: PLoS Genet. 2008 Apr 11;4(4):e1000052. doi: 10.1371/journal.pgen.1000052 (PMC2290968; doi:10.1371/journal.pgen.1000052)

Figure S4. **Structure analysis of genotypic variation**. Each individual is represented by a column that is partitioned into *K* colored segments representing the proportion of ancestry (*Q* value) from each of the *K* clusters for each individual using 11,000 autosomal SNP markers. Two Structure run at *K* = 2 and *K* = 3 are shown. At *K* = 3, 80% of individuals have high membership coefficient to one cluster.

***K* = 2**

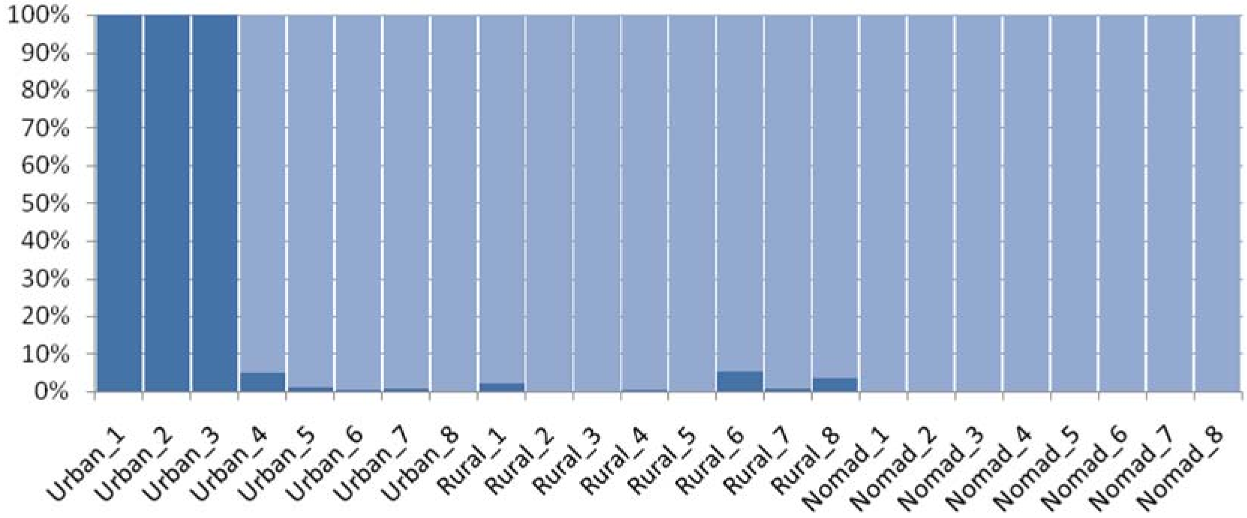

***K* = 3**

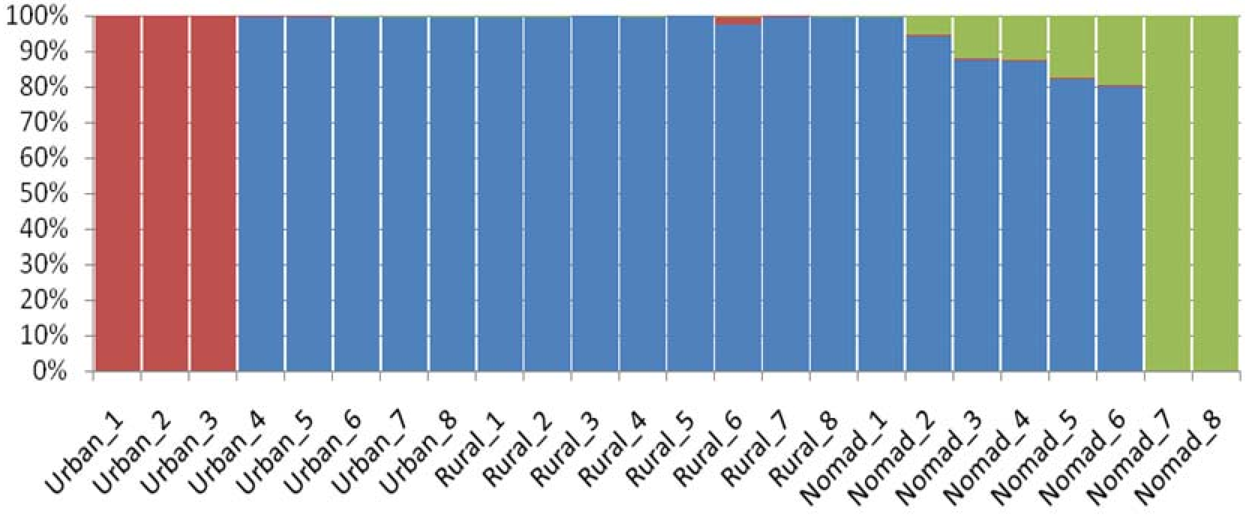

Supplement: Figure S4 — Structure analysis of genotypic variation. Each individual is represented by a column that is partitioned into K colored segments representing the proportion of ancestry (Q value) from each of the K clusters for each individual using 11,000 autosomal SNP markers. Two Structure run at K = 2 and K = 3 are shown. At K = 3, 80% of individuals have high membership coefficient to one cluster. (0.15 MB DOC) [file pgen.1000052.s004.pdf]
