# Supplementary material for: A Genome-Wide Gene Expression Signature of Environmental Geography in Leukocytes of Moroccan Amazighs
Source: PLoS Genet. 2008 Apr 11;4(4):e1000052. doi: 10.1371/journal.pgen.1000052 (PMC2290968; doi:10.1371/journal.pgen.1000052)

Figure S5. Genomewide genetic and gene expression differentiation. Average *Fst* measures plotted against log2 fold change (**A**) and negative log10 probability resulting from differential expression analyses with SVA (**B**), for each of the 10,177 expressed genes and for each population pairwise comparison (Green, Nomadic vs. Rural; Blue, Nomadic vs. Urban; Red, Urban vs. Rural). Each open circle represents a gene. Gene-specific *Fst* values were calculated for each gene by averaging *Fst* values for all segregating SNPs located within 1-Mb upstream and downstream of the expression probe. The percent variance explained is less than 0.001 with $p > 0.05$ for all six regressions represented by these plots.

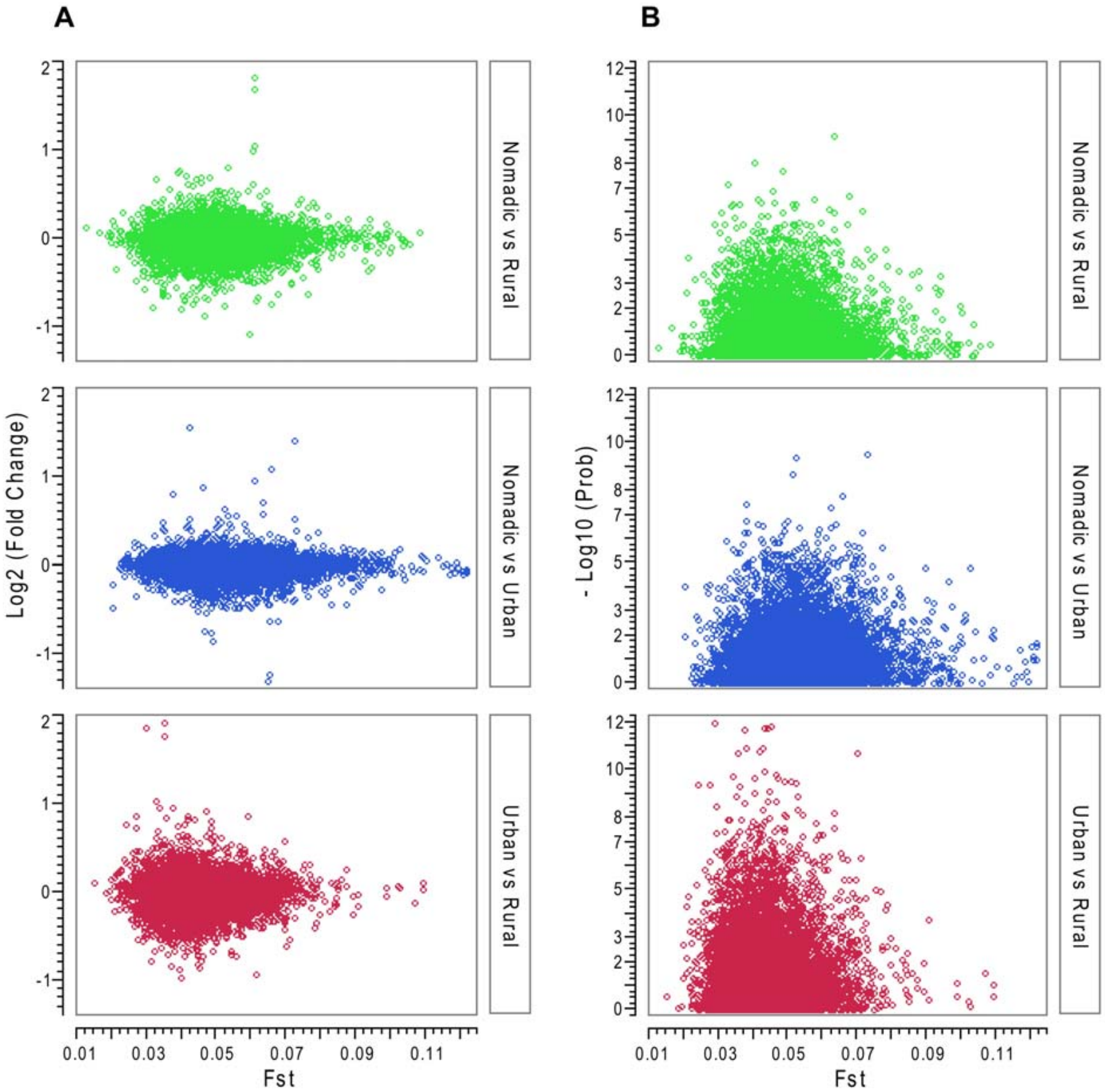

Supplement: Figure S5 — Genomewide genetic and gene expression differentiation. Average Fst measures plotted against log2 fold change (A) and negative log10 probability resulting from differential expression analyses with SVA (B), for each of the 10,177 expressed genes and for each population pairwise comparison (Green, Nomadic vs. Rural; Blue, Nomadic vs. Urban; Red, Urban vs. Rural). Each open circle represents a gene. Gene-specific Fst values were calculated for each gene by averaging Fst values for all segregating SNPs within the gene and approximately 1Mb upstream and downstream the gene. The percent variance explained is less than 0.001 with p>0.05 for all six regressions represented by these plots. (0.17 MB PDF) [file pgen.1000052.s005.pdf]
