# Supplementary material for: A Genome-Wide Gene Expression Signature of Environmental Geography in Leukocytes of Moroccan Amazighs
Source: PLoS Genet. 2008 Apr 11;4(4):e1000052. doi: 10.1371/journal.pgen.1000052 (PMC2290968; doi:10.1371/journal.pgen.1000052)

Figure S6. **Results of methylation analysis**. Histograms show the distribution by chromosome of CpG sites of (A) the 1,505 CpG sites represented on Illumina's GoldenGate Methylation Cancer Panel I array; (B) the 97 differentially methylated CpG sites for the sex effect at $P < 0.05$ (ANOVA); (C) the 69 differentially methylated CpG sites between locations at $P < 0.05$, and (D) the 24 differentially methylated CpG sites for the sex and location interaction effect at $P < 0.05$. The X chromosome is shown in dark green. Panel B can be considered a positive control for the success of the analysis since methylation is known to preferentially mark X-linked loci.

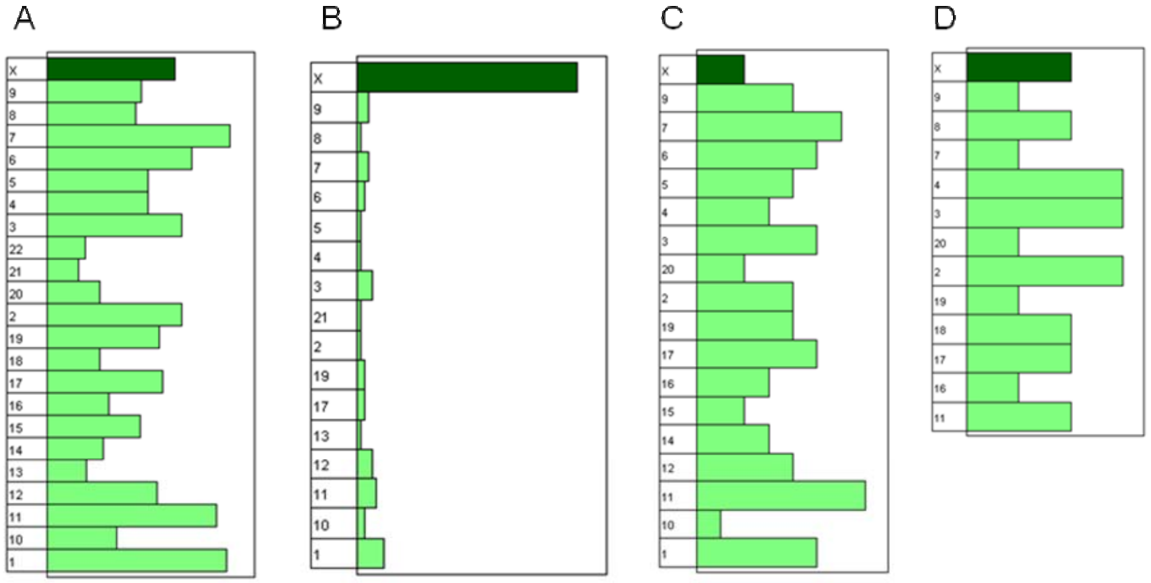

Supplement: Figure S6 — Results of methylation analysis. Histograms show the distribution by chromosome of CpG sites of (A) the 1,505 CpG sites represented on Illumina's GoldenGate Methylation Cancer Panel I array; (B) the 97 differentially methylated CpG sites for the sex effect at P<0.05 (ANOVA); (C) the 69 differentially methylated CpG sites between locations at P<0.05, and (D) the 24 differentially methylated CpG sites for the sex and location interaction effect at P<0.05. The X chromosome is shown in dark green. Panel B can be considered a positive control for the success of the analysis since methylation is known to preferentially mark X-linked loci. (0.14 MB PDF) [file pgen.1000052.s006.pdf]
