# Supplementary material for: A Genome-Wide Gene Expression Signature of Environmental Geography in Leukocytes of Moroccan Amazighs
Source: PLoS Genet. 2008 Apr 11;4(4):e1000052. doi: 10.1371/journal.pgen.1000052 (PMC2290968; doi:10.1371/journal.pgen.1000052)

Figure S7. **Correlations among Interleukin signaling components**. The three plots show the average relative transcript abundance (log base 2 scale) for the indicated genes in the Urban (U: Anza), Nomadic (N: Bedouin), and Rural (R: Sebt-Nabor) locations. *P* values in brackets indicate the significance of the 3-way location term from mixed model ANOVA. Across all 46 individuals, IL-8 is negatively correlated with IRAK2 ($P = 0.0008$), IRAK1 is negatively correlated with IRAKBP1 ($P = 0.008$), and IRAK3 is positively correlated with IRAK4 ($P = 0.004$).

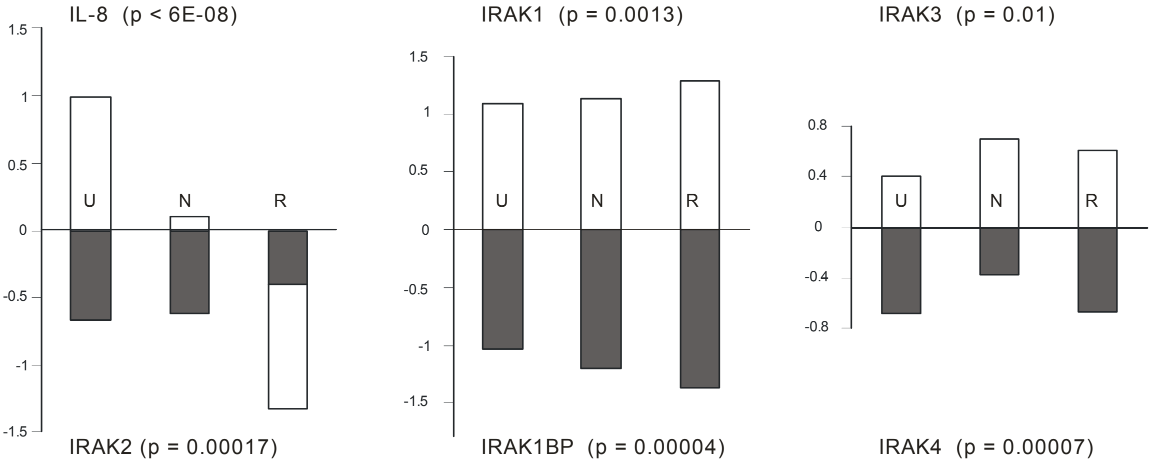

Supplement: Figure S7 — Correlations among Interleukin signaling components. The three plots show the average relative transcript abundance (log base 2 scale) for the indicated genes in the Urban (U: Anza), Nomadic (N: Bedouin), and Rural (R: Sebt-Nabor) locations. P values in brackets indicate the significance of the 3-way location term from mixed model ANOVA. Across all 46 individuals, IL-8 is negatively correlated with IRAK2 (P = 0.0008), IRAK1 is negatively correlated with IRAKBP1 (P = 0.008), and IRAK3 is positively correlated with IRAK4 (P = 0.004). (0.08 MB PDF) [file pgen.1000052.s007.pdf]
