# Supplementary material for: A Genome-Wide Gene Expression Signature of Environmental Geography in Leukocytes of Moroccan Amazighs
Source: PLoS Genet. 2008 Apr 11;4(4):e1000052. doi: 10.1371/journal.pgen.1000052 (PMC2290968; doi:10.1371/journal.pgen.1000052)
Supplement: Table S2 — Eigenstrat principal component statistics (0.06 MB PDF) [file pgen.1000052.s009.pdf]

Table S2. ***Eigenstrat* principal component statistics**. The program uses unsupervised Tracy-Widom (TW) statistics to test if the means of the eigenvector coordinates associated to each individual in each population differ significantly.

Eigenvector	Eigenvalue	TW Statistic	TW *P*-value
1	2.005631	3.503	0.000627
2	1.666154	1.709	0.016769
3	1.389458	-1.513	0.577793
4	1.201761	-4.663	0.999032
5	1.148618	-5.237	0.999895
6	1.108053	-5.567	0.999976
7	1.069332	-5.872	0.999995
8	1.054077	-5.647	0.999984
9	1.035545	-5.468	0.999962
10	1.016427	-5.278	0.999911
