# Supplementary material for: A Genome-Wide Gene Expression Signature of Environmental Geography in Leukocytes of Moroccan Amazighs
Source: PLoS Genet. 2008 Apr 11;4(4):e1000052. doi: 10.1371/journal.pgen.1000052 (PMC2290968; doi:10.1371/journal.pgen.1000052)
Supplement: Table S3 — List of genes in FOS and MYC networks shown in Figure 4A (0.09 MB PDF) [file pgen.1000052.s010.pdf]

Table S3. **List of genes in FOS and MYC networks shown in Figure 4A**. Fold change and *P* value from surrogate variable analysis followed by mixed model analysis of variance as described in material and methods are shown.

Name	Entrez_ID	Fold Change	*P*-value	Location
ACTG1	71	-1.5294026	0.00605659	Cytoplasm
ALPP	250	-1.278339	0.00114771	Extracell. Space
ANKS1A	23294	-1.1743818	2.05E-05	Cytoplasm
ANXA2	302	-1.1844711	0.00299349	Plasma Memb.
ARL6IP1	23204	-1.1324935	0.00119596	Cytoplasm
BAT1	7919	1.34768336	4.91E-04	Nucleus
BOP1	23246	-1.3220285	3.00E-09	Nucleus
CDR2	1039	1.21897037	3.18E-05	Cytoplasm
CHMP6	79643	-1.2052507	1.29E-05	Cytoplasm
COTL1	23406	-1.1483082	2.16E-04	Cytoplasm
CPD	1362	-1.3318136	1.40E-06	Extracell. Space
EAF1	85403	-1.1383807	6.71E-04	Nucleus
ELL	8178	-1.3741125	2.04E-06	Nucleus
EMB	133418	1.31891314	1.70E-05	Plasma Memb.
FARS2	10667	1.10651023	0.00228784	Cytoplasm
FOS	2353	3.67294184	2.47E-05	Nucleus
GAK	2580	-1.3105464	5.70E-09	Nucleus
GM2A	2760	-1.6517571	0.00184158	Cytoplasm
H3F3A	3020	1.07691646	0.00331633	Nucleus
H3F3B	3021	1.4959445	1.27E-04	Nucleus
HGS	9146	-1.2682638	6.41E-07	Cytoplasm
HIVEP2	3097	1.10719351	0.0021508	Nucleus
LRSAM1	90678	-1.3993772	1.00E-10	Cytoplasm
MFAP1	4236	-1.0909704	7.20E-04	Extracell. Space
MGST3	4259	-1.2119304	5.90E-04	Cytoplasm
MYC	4609	1.16830351	1.94E-04	Nucleus
NOL5A	10528	1.30772704	2.78E-04	Nucleus
NOP5/NOP58	51602	1.27398178	1.40E-08	Nucleus
PA2G4	5036	1.2514751	1.10E-05	Nucleus
PARP10	84875	-1.3587708	1.04E-05	Nucleus
POLE	5426	-1.2175069	1.43E-05	Nucleus
PPP1R16A	84988	-1.0816162	0.0036466	Plasma Memb.
PRDX3	10935	-1.1246358	0.00294685	Cytoplasm
RFX1	5989	-1.1928743	2.74E-04	Nucleus
RNH1	6050	-1.1262036	0.00559963	Cytoplasm

RPL12	6136	1.42007109	1.00E-11	Cytoplasm
RPL19	6143	1.14053192	5.94E-05	Cytoplasm
RPL27	6155	1.19911635	1.85E-05	Cytoplasm
RPL30	6156	1.3444361	2.00E-10	Cytoplasm
RPL34	6164	1.17708518	3.89E-06	Cytoplasm
RPL35	11224	1.31008932	7.38E-08	Cytoplasm
RPS12	6206	1.14229077	1.35E-05	Cytoplasm
RPS18	6222	1.24545327	3.57E-04	Cytoplasm
RPS20	6224	1.30484429	2.11E-05	Cytoplasm
RPS7	6201	1.18451853	7.56E-06	Cytoplasm
RPS9	6203	1.15786488	0.0017371	Cytoplasm
SCPEP1	59342	-1.2233267	1.30E-04	Cytoplasm
SEC23B	10483	-1.2177539	3.22E-07	Cytoplasm
SIAHBP1	22827	-1.2650019	5.09E-07	Nucleus
SLC11A1	6556	-1.4352495	3.60E-04	Plasma Memb.
SLC25A19	60386	-1.1533171	3.83E-04	Cytoplasm
SLC25A5	292	-1.1427435	0.00131215	Cytoplasm
SMARCD2	6603	-1.2446635	1.44E-06	Nucleus
SNF8	11267	-1.4052659	3.75E-05	Nucleus
SPTBN1	6711	1.24295752	0.00198691	Plasma Memb.
SQRDL	58472	-1.1583783	0.00199505	Cytoplasm
STX12	23673	-1.1135745	7.53E-04	Plasma Memb.
TCEA1	6917	1.19374442	1.94E-07	Nucleus
TGFBI	7045	-1.417562	0.00114392	Extracell. Space
TSG101	7251	1.13423903	1.15E-04	Nucleus
TSPO	706	-1.3148237	4.99E-06	Cytoplasm
VDAC1	7416	-1.1159642	0.00303239	Cytoplasm
VDAC2	7417	-1.1526311	3.15E-04	Cytoplasm
VPS36	51028	1.21219122	0.00438165	Cytoplasm
VPS37C	55048	-1.2345254	0.00359611	Cytoplasm
ZNF33A	7581	1.09598622	0.00125708	Nucleus
ZNF467	168544	-1.3810602	1.51E-04	Nucleus
