# Supplementary material for: A Genome-Wide Gene Expression Signature of Environmental Geography in Leukocytes of Moroccan Amazighs
Source: PLoS Genet. 2008 Apr 11;4(4):e1000052. doi: 10.1371/journal.pgen.1000052 (PMC2290968; doi:10.1371/journal.pgen.1000052)
Supplement: Table S4 — Top 15 networks generated by Ingenuity from the list of genes differentially expressed (1% FDR) for each location pairwise comparison (0.11 MB PDF) [file pgen.1000052.s011.pdf]

Table S4. **Top 15 networks generated by Ingenuity from the list of genes differentially expressed (1% FDR) for each location pairwise comparison**. The genes called Focus Genes are genes that meet the 1% FDR cutoff in addition to the presence of at least one other full-length, wild-type gene or protein in the IPKB that interacts (directly or indirectly) with these genes. The networks are sorted by their score that is from the likelihood that the Focus Genes that are part of a network are found therein by random chance alone.

Network	Analysis	Score	Focus Genes	Top Functions
1	Nomadic_vs_Rural	43	30	Cell Cycle, Cancer, Gastrointestinal Disease
1	Nomadic_vs_Urban	41	31	Protein Synthesis, Cancer, Cell Cycle
1	Rural_vs_Urban	38	35	RNA Post-Transcriptional Modification, Cellular Assembly and Organization, Protein Synthesis
2	Nomadic_vs_Urban	41	31	Cancer, Cell Cycle, Reproductive System Disease
2	Nomadic_vs_Rural	40	29	Gene Expression, Cell Morphology, Connective Tissue Development and Function
2	Rural_vs_Urban	35	34	Cell Morphology, Cellular Assembly and Organization, Cellular Development
3	Nomadic_vs_Urban	39	30	Cellular Development, Post-Translational Modification, Cellular Growth and Proliferation
3	Nomadic_vs_Rural	36	27	Cellular Assembly and Organization, Cancer, Cell Death
3	Rural_vs_Urban	35	34	Viral Function, Cellular Function and Maintenance, Post-Translational Modification
4	Nomadic_vs_Urban	37	29	Nucleic Acid Metabolism, Gene Expression, Cancer
4	Nomadic_vs_Rural	36	27	Cancer, Cellular Growth and Proliferation, Cell Signaling
4	Rural_vs_Urban	35	34	Protein Degradation, Gene Expression, DNA Replication, Recombination, and Repair
5	Nomadic_vs_Urban	37	29	Cell Signaling, Immune Response, Carbohydrate Metabolism
5	Nomadic_vs_Rural	34	26	Vitamin and Mineral Metabolism, Lipid Metabolism, Cancer
5	Rural_vs_Urban	33	33	Cellular Development, Reproductive System Development and Function, Cellular Assembly and Organization
6	Nomadic_vs_Urban	37	29	Cancer, Cell Death, Reproductive System Disease
6	Rural_vs_Urban	33	33	Immune Response, Hematological System Development and Function, Cellular Development
6	Nomadic_vs_Rural	30	24	Post-Translational Modification, Gene Expression, Cell Cycle
7	Nomadic_vs_Urban	35	28	DNA Replication, Recombination, and Repair, Cell Cycle, Cellular Assembly and Organization

7	Rural_vs_Urban	33	33	Cardiovascular System Development and Function, Protein Degradation, Cell Signaling
7	Nomadic_vs_Rural	28	23	Cell-To-Cell Signaling and Interaction, Cellular Assembly and Organization, Hematological System Development and Function
8	Nomadic_vs_Urban	35	28	Molecular Transport, Protein Trafficking, Cell Death
8	Rural_vs_Urban	31	32	RNA Post-Transcriptional Modification, Cellular Assembly and Organization, Carbohydrate Metabolism
8	Nomadic_vs_Rural	26	22	Cancer, Hematological Disease, Cellular Development
9	Nomadic_vs_Urban	35	28	Protein Synthesis, Gene Expression, Cell Cycle
9	Rural_vs_Urban	31	32	Cellular Assembly and Organization, Vitamin and Mineral Metabolism, Lipid Metabolism
9	Nomadic_vs_Rural	19	18	Immune Response, Neurological Disease, Cardiovascular System Development and Function
10	Nomadic_vs_Urban	33	27	Hematological System Development and Function, Immune and Lymphatic System Development and Function
10	Rural_vs_Urban	31	32	Gene Expression, Cardiovascular System Development and Function, Cell-To-Cell Signaling and Interaction
10	Nomadic_vs_Rural	18	17	Cancer, Reproductive System Disease, Renal and Urological Disease
11	Nomadic_vs_Urban	27	24	Cell Signaling, Tissue Development, Cardiovascular System Development and Function
11	Nomadic_vs_Rural	18	17	Ophthalmic Disease, Cellular Function and Maintenance, Cellular Assembly and Organization
12	Rural_vs_Urban	31	32	Cardiovascular Disease, Metabolic Disease, RNA Post-Transcriptional Modification
12	Nomadic_vs_Urban	27	24	Cardiovascular System Development and Function, Tissue Morphology, Lipid Metabolism
12	Nomadic_vs_Rural	16	16	Lipid Metabolism, Small Molecule Biochemistry, Molecular Transport
13	Rural_vs_Urban	31	32	Cell Signaling, Gene Expression, Cancer
13	Nomadic_vs_Urban	25	23	Cellular Assembly and Organization, Cellular Movement, Gene Expression
13	Nomadic_vs_Rural	16	16	Cell Signaling, Immune Response, Hematological System Development and Function
14	Rural_vs_Urban	31	32	Gene Expression, Carbohydrate Metabolism, Cancer
14	Nomadic_vs_Urban	19	19	Immune Response, Cancer, Tumor Morphology
14	Nomadic_vs_Rural	16	16	Cancer, Cell Cycle, Gene Expression
15	Rural_vs_Urban	29	31	Cellular Growth and Proliferation, Hematological System Development and Function, Immune Response
15	Nomadic_vs_Rural	16	16	Dermatological Diseases and Conditions, Gene Expression, Cell Signaling
15	Nomadic_vs_Urban	14	16	Cellular Development, Cellular Growth and Proliferation, Reproductive System Disease
