# Supplementary material for: A Genome-Wide Gene Expression Signature of Environmental Geography in Leukocytes of Moroccan Amazighs
Source: PLoS Genet. 2008 Apr 11;4(4):e1000052. doi: 10.1371/journal.pgen.1000052 (PMC2290968; doi:10.1371/journal.pgen.1000052)
Supplement: Table S5 — List of individuals and analyses (0.09 MB PDF) [file pgen.1000052.s012.pdf]

Table S5. List of individuals and analyses. 52 unique individuals were samples from the three lifestyles shown in the table. Samples used for each experiment (gene expression, genotyping, and methylation) as well as the samples that were replicated are also shown.

#	Sample_ID	Expression	Exp_ID	Array_ID	Batch	Genotype	Methylation	Sex	Lifestyle
1	A03	YES	FA8B	1743113118_C	A	YES	YES	FEM	URBAN
2	A09	YES	MA8D	1743113118_E	A	YES	YES	MAL	URBAN
3	A11	YES	MA9C	1743113119_C	A	YES	YES	MAL	URBAN
4	A12	YES	FA9F	1743113119_G	A	NO	YES	FEM	URBAN
5	A16	YES	MA4A	1743113134_A	A	YES	YES	MAL	URBAN
6	A18	YES	FA4C	1743113134_C	A	NO	YES	FEM	URBAN
7	A15	YES	MA4D	1743113134_D	A	YES	YES	MAL	URBAN
8	A14	YES	MA4E	1743113134_E	A	NO	YES	MAL	URBAN
9	A8	YES	FA6C	1743113136_D	A	NO	YES	FEM	URBAN
10	A6	YES	FA6D	1743113136_E	A	NO	YES	FEM	URBAN
11	A17	YES	MA2E	1743113062_E	B	YES	YES	MAL	URBAN
12	A22	YES	MA2G	1743113062_G	B	YES	YES	MAL	URBAN
13	A21	YES	MA3B	1743113063_B	B	NO	YES	MAL	URBAN
14	A01	YES	MA3C	1743113063_C	B	YES	YES	MAL	URBAN
15	A13	YES	FA3E	1743113063_E	B	NO	YES	FEM	URBAN
16	A07	YES	FA5D	1743113076_D	B	NO	YES	FEM	URBAN
17	A19	YES	MA5G	1743113076_G	B	NO	YES	MAL	URBAN
18	A20	YES	MA7E	1743113077_E	B	NO	YES	MAL	URBAN
19	E13	YES	MD8A	1743113118_A	A	YES	YES	MAL	NOMAD
20	E10	YES	MD8E	1743113118_F	A	NO	YES	MAL	NOMAD
21	E09	YES	MD9A	1743113119_E	A	YES	YES	MAL	NOMAD
22	E01	YES	MD9E	1743113119_H	A	YES	YES	MAL	NOMAD
23	E12	YES	FD4B	1743113134_B	A	YES	YES	FEM	NOMAD
24	E6	YES	FD4H	1743113134_H	A	NO	YES	FEM	NOMAD
25	E11	YES	FD6E	1743113136_F	A	NO	YES	FEM	NOMAD
26	E14	YES	MD6F	1743113136_H	A	YES	YES	MAL	NOMAD
27	E05	YES	MD7H	1743113077_H	B	YES	YES	MAL	NOMAD
28	E03	YES	FD2D	1743113062_D	B	NO	NO	FEM	NOMAD
29	E17	YES	MD2F	1743113062_F	B	YES	YES	MAL	NOMAD
30	E07	YES	FD2H	1743113062_H	B	NO	YES	FEM	NOMAD
31	E16	YES	FD3A	1743113063_A	B	NO	YES	FEM	NOMAD
32	E04	YES	MD3H	1743113063_H	B	YES	YES	MAL	NOMAD

33	E08	YES	FD5H	1743113076_H	B	NO	NO	FEM	NOMAD
34	E15	YES	MD7F	1743113077_F	B	NO	YES	MAL	NOMAD
35	SN1	YES	FV8C	1743113118_D	A	YES	YES	FEM	RURAL
36	SN10	YES	FV8F	1743113118_H	A	NO	YES	FEM	RURAL
37	SN16	YES	MV9B	1743113119_B	A	YES	YES	MAL	RURAL
38	SN3	YES	FV9D	1743113119_D	A	NO	YES	FEM	RURAL
39	SN17	YES	MV4A	1743113134_F	A	YES	YES	MAL	RURAL
40	SN18	YES	MV4G	1743113134_G	A	YES	YES	MAL	RURAL
41	SN12	YES	FV6A	1743113136_A	A	NO	YES	FEM	RURAL
42	SN2	YES	FV6B	1743113136_C	A	NO	YES	FEM	RURAL
43	SN13	YES	MV2B	1743113062_B	B	YES	YES	MAL	RURAL
44	SN11	YES	FV3G	1743113063_G	B	NO	YES	FEM	RURAL
45	SN14	YES	MV7A	1743113077_A	B	YES	YES	MAL	RURAL
46	SN7	YES	FV7B	1743113077_B	B	YES	YES	FEM	RURAL
47	SN5	POOR	---	1743113077_G	B	YES	YES	FEM	RURAL
48	SN15	POOR	---	1743113076_E	B	NO	YES	MAL	RURAL
49	A4	POOR	---	1743113077_C	B	NO	YES	MAL	URBAN
50	A23	POOR	---	1743113076_B	B	NO	NO	MAL	URBAN
51	E2	POOR	---	1743113076_C	B	NO	NO	MAL	NOMAD
52	A08_rep	POOR	FA6C	1743113076_F	B	NO	YES	FEM	URBAN
53	SN17_rep	YES	MV3F	1743113063_F	B	YES	YES	MAL	RURAL
54	SN13_rep	YES	MV3D	1743113063_D	B	YES	YES	MAL	RURAL
55	E03_rep	YES	FD5A	1743113076_A	B	NO	NO	FEM	NOMAD
56	E05_rep	YES	MD2A	1743113062_A	B	YES	YES	MAL	NOMAD
57	SN8	NO	---	---		NO	YES	FEM	RURAL
